# Assessing the Influence of COVID-19 Vaccination Coverage on Excess Mortality across 178 Countries: A Cross-Sectional Study

**DOI:** 10.3390/vaccines11081294

**Published:** 2023-07-28

**Authors:** Oliver Mendoza-Cano, Xóchitl Trujillo, Miguel Huerta, Mónica Ríos-Silva, José Guzmán-Esquivel, Agustin Lugo-Radillo, Verónica Benites-Godínez, Jaime Alberto Bricio-Barrios, Martha Irazema Cárdenas-Rojas, Eder Fernando Ríos-Bracamontes, Hannah Priscila Guzman-Solorzano, Greta Mariana Baltazar-Rodríguez, Valeria Ruiz-Montes de Oca, Vannya Marisol Ortega-Macías, Ana Daniela Ortega-Ramírez, Efrén Murillo-Zamora

**Affiliations:** 1 Facultad de Ingeniería Civil, Universidad de Colima, km. 9 Carretera Colima-Coquimatlán, Colima 28400, Mexico; 2 Centro Universitario de Investigaciones Biomédicas, Universidad de Colima, Av. 25 de Julio 965, Col. Villas San Sebastián, Colima 28045, Mexico; 3 Centro Universitario de Investigaciones Biomédicas, CONAHCyT—Universidad de Colima, Av. 25 de Julio 965, Col. Villas San Sebastián, Colima 28045, Mexico; 4 Unidad de Investigación en Epidemiología Clínica, Instituto Mexicano del Seguro Social, Av. Lapislázuli 250, Col. El Haya, Colima 28984, Mexico; 5 CONAHCyT—Facultad de Medicina y Cirugía, Universidad Autónoma Benito Juárez de Oaxaca, Ex Hacienda Aguilera S/N, Carr. A San Felipe del Agua, Oaxaca 68020, Mexico; 6 Coordinación de Educación en Salud, Instituto Mexicano del Seguro Social, Calzada del Ejercito Nacional 14, Col. Fray Junípero Serra, Nayarit 63160, Mexico; 7 Unidad Académica de Medicina, Universidad Autónoma de Nayarit, Ciudad de la Cultura Amado Nervo, Nayarit 63155, Mexico; 8 Facultad de Medicina, Universidad de Colima, Av. Universidad 333, Col. Las Víboras, Colima 28040, Mexico; 9 Departamento de Medicina Interna, Hospital General de Zona No. 1, Instituto Mexicano del Seguro Social, Av. Lapislázuli 250, Col. El Haya, Colima 28984, Mexico; 10 Instituto Tecnológico y de Estudios Superiores de Monterrey, Escuela de Medicina y Ciencias de la Salud, Campus Guadalajara, Av. Gral. Ramón Corona No. 2514, Colonia Nuevo México, Zapopan 45201, Mexico; 11 Plantel Guadalajara, Escuela de Medicina, Universidad Cuauhtémoc, Av. del Bajío No. 5901, Col. Del Bajío, Zapopan 45019, Mexico; 12 Programa de Doctorado en Ciencias Médicas, Facultad de Medicina, Universidad de Colima, Av. Universidad 333, Col. Las Víboras, Colima 28040, Mexico

**Keywords:** COVID-19, pandemics, mortality, vaccination coverage

## Abstract

The COVID-19 pandemic has had a devastating impact on global health, necessitating urgent and effective strategies to mitigate its consequences. Vaccination programs have been implemented worldwide to combat virus transmission and reduce the disease burden. This study aimed to investigate the relationship between COVID-19 vaccination coverage and all-cause excess mortality in 178 nations during the first two years of the pandemic. Multiple regression analysis, after adjusting for life expectancy at birth, confirmed a significant association between higher vaccination coverage and lower all-cause mortality rates (β = −106.8, 95% CI −175.4 to −38.2, p = 0.002). These findings underscore the importance of vaccination campaigns in reducing overall mortality during the COVID-19 pandemic. Evidence-based decision making and resource allocation can benefit from this information, facilitating the optimization of vaccination strategies for maximal impact on mortality reduction. Further research and continuous monitoring are crucial to understanding the long-term effects of vaccination coverage on population health in the ongoing pandemic.

## 1. Introduction

The COVID-19 pandemic has unleashed an unprecedented wave of morbidity and mortality worldwide, affecting millions of lives and overwhelming healthcare systems. In response, governments and healthcare authorities around the world have developed and distributed COVID-19 vaccines, implementing vaccination programs as a fundamental strategy to combat the transmission of the virus and alleviate the burden of the disease [1,2]. Investigating the impact of vaccination coverage on overall mortality rates is a crucial area of research [3].

The evaluation of all-cause excess mortality during the COVID-19 pandemic is a comprehensive approach that allows for a holistic understanding of the pandemic’s impact beyond the direct effects of the virus. Excess mortality, defined as the difference between observed and expected deaths, provides insights into the indirect consequences of the pandemic, such as disruptions in healthcare systems, delays in non-COVID-related treatments, and the socioeconomic impact on vulnerable populations [4]. Assessing the correlation between COVID-19 vaccination coverage and all-cause excess mortality provides valuable information on the overall effectiveness of vaccination campaigns and their potential to mitigate the broader health consequences of the pandemic.

This study aimed to analyze and evaluate the relationship between all-cause excess mortality during the first two years of the COVID-19 pandemic (2020–2021) and COVID-19 vaccination coverage. We hypothesize that countries with lower immunization coverage experienced higher rates of excess mortality. This adverse scenario may result from a combination of factors, including increased transmission and higher infection rates due to a larger proportion of the susceptible population [5], and challenges in implementing effective public health measures and mitigation strategies [6], among others.

By employing comprehensive datasets, we sought to identify the potential impact of vaccination coverage on overall mortality trends across nations. This evaluation could guide evidence-based decision-making processes, facilitate resource allocation, and inform future public health strategies aimed at optimizing vaccination campaigns to maximize their impact on reducing mortality.

## 2. Materials and Methods

A cross-sectional analysis was performed on publicly available datasets using an ecological approach. All analyzed datasets were accessed and consulted on 15 May 2023.

### 2.1. Data Sources

The rates of excess mortality for all causes (per 100,000) associated with the COVID-19 pandemic from 2020 to 2021 were obtained from the World Health Organization (WHO) website (https://www.who.int/data/sets/global-excess-deaths-associated-with-covid-19-modelled-estimates (accessed on 15 May 2023). These calculations were derived using standardized methods for surplus mortality, which involved establishing a consistent and comparable methodology to calculate excess mortality across various regions and time periods, taking into account factors such as age, gender, and cause of death [7].

The coverage of COVID-19 vaccinations, measured as the percentage of the population that received at least one dose of any COVID-19 vaccine, was obtained from the Coronavirus Resource Center at Johns Hopkins University website (https://coronavirus.jhu.edu/vaccines/international (accessed on 15 May 2023). The analyzed data included information accumulated until 10 March 2023.

To adjust our models for unobserved variables that may influence all-cause excess mortality, we incorporated life expectancy at birth as a relevant variable. We utilized the most recent available estimates (corresponding to 2021) from the World Bank website (https://api.worldbank.org/v2/en/indicator/SP.DYN.LE00.IN?downloadformat=csv (accessed on 15 May 2023). Life expectancies for both sexes combined were utilized in our analysis. These estimates are derived from the latest revision (2022) of the World Population Prospects [8].

Countries that lacked any of the three variables after merging all the databases were excluded from the analysis. A total of 178 nations from all continents were included and the complete list was as follows: Afghanistan, Albania, Algeria, Angola, Antigua and Barbuda, Argentina, Armenia, Australia, Austria, Azerbaijan, Bahamas, Bahrain, Bangladesh, Barbados, Belarus, Belgium, Belize, Benin, Bhutan, Bolivia, Bosnia and Herzegovina, Botswana, Brazil, Brunei Darussalam, Bulgaria, Burkina Faso, Burundi, Cabo Verde, Cambodia, Cameroon, Canada, Central African Republic, Chad, Chile, China, Colombia, Comoros, Congo, Costa Rica, Croatia, Cuba, Cyprus, Czechia, Côte d’Ivoire, Democratic People’s Republic of Korea, Democratic Republic of the Congo, Denmark, Djibouti, Dominican Republic, Ecuador, Egypt, El Salvador, Equatorial Guinea, Estonia, Eswatini, Ethiopia, Fiji, Finland, France, Gabon, Gambia, Georgia, Germany, Ghana, Greece, Grenada, Guatemala, Guinea, Guinea-Bissau, Guyana, Haiti, Honduras, Hungary, Iceland, India, Indonesia, Iran, Iraq, Ireland, Israel, Italy, Jamaica, Japan, Jordan, Kazakhstan, Kenya, Kiribati, Kuwait, Kyrgyzstan, Lao People’s Democratic Republic, Latvia, Lebanon, Lesotho, Liberia, Libya, Lithuania, Luxembourg, Madagascar, Malawi, Malaysia, Maldives, Mali, Malta, Mauritania, Mauritius, Mexico, Micronesia, Mongolia, Montenegro, Morocco, Mozambique, Namibia, Nepal, Netherlands, New Zealand, Nicaragua, Niger, Nigeria, North Macedonia, Norway, Oman, Pakistan, Panama, Papua New Guinea, Paraguay, Peru, Philippines, Poland, Portugal, Qatar, Republic of Moldova, Romania, Russian Federation, Rwanda, Saint Lucia, Saint Vincent and the Grenadines, Samoa, Sao Tome and Principe, Saudi Arabia, Senegal, Serbia, Seychelles, Sierra Leone, Singapore, Slovakia, Slovenia, Solomon Islands, Somalia, South Africa, South Sudan, Spain, Sri Lanka, Sudan, Suriname, Sweden, Switzerland, Syrian Arab Republic, Tajikistan, Thailand, The United Kingdom, Timor-Leste, Togo, Trinidad and Tobago, Tunisia, Turkey, Uganda, Ukraine, United Arab Emirates, United Republic of Tanzania, United States of America, Uruguay, Uzbekistan, Vanuatu, Venezuela, Vietnam, Yemen, Zambia, and Zimbabwe.

### 2.2. Statistical Analysis

We calculated summary statistics and Spearman’s correlation coefficients (rho), along with 95% confidence intervals (CI), to assess the association between the ecological variables of interest. Lastly, a multiple linear regression model was constructed to estimate regression coefficients (β) and 95% CI. The assumptions of linear regression were assessed to ensure the appropriateness of using it for evaluating the outcome of interest.

### 2.3. Ethical Considerations

We analyzed publicly available and fully deidentified datasets, thereby exempting the need for ethics approval in health research. Nevertheless, all aspects of this study adhered to strict international ethics guidelines.

## 3. Results

### 3.1. All-Cause Excess Mortality Rate

**Figure 1:**
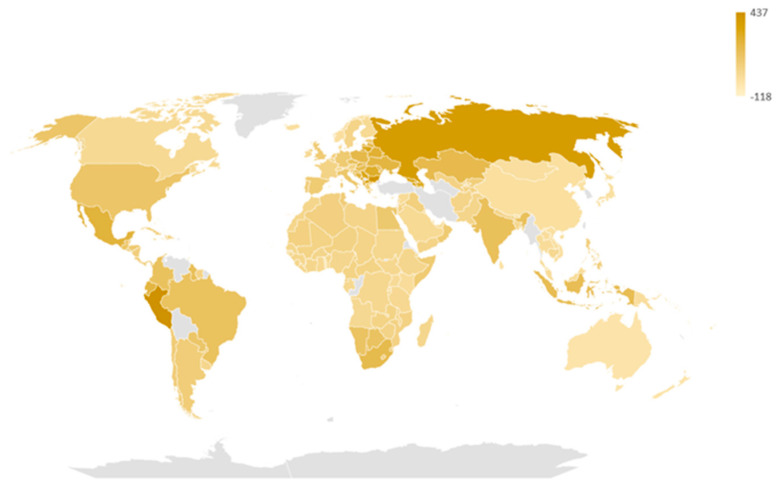
All-cause excess mortality rate (per 100,000) from 2020 to 2021 in the 178 analyzed countries.

The median all-cause excess mortality rate among the 178 analyzed nations was 63 per 100,000, with an interquartile range spanning from 33 to 131 per 100,000. In Mongolia, the observed mortality rate aligned with expectations, resulting in a corresponding rate of 0. Out of the total, 21 countries (Antigua and Barbuda, Australia, Barbados, Bhutan, Brunei Darussalam, China, Republic of Korea, Fiji, Grenada, Iceland, Japan, Kiribati, Micronesia, New Zealand, Norway, Samoa, Solomon Islands, Sri Lanka, Togo, Vanuatu, and Vietnam) did not exhibit any positive all-cause excess mortality. Among the remaining nations with positive all-cause excess mortality (n = 156), the rates ranged from 4 to 437 per 100,000 in Seychelles and Peru, respectively. Figure 1 presents a choropleth map illustrating the all-cause excess mortality rate in all of the analyzed nations.

### 3.2. COVID-19 Vaccination Coverage

**Figure 2:**
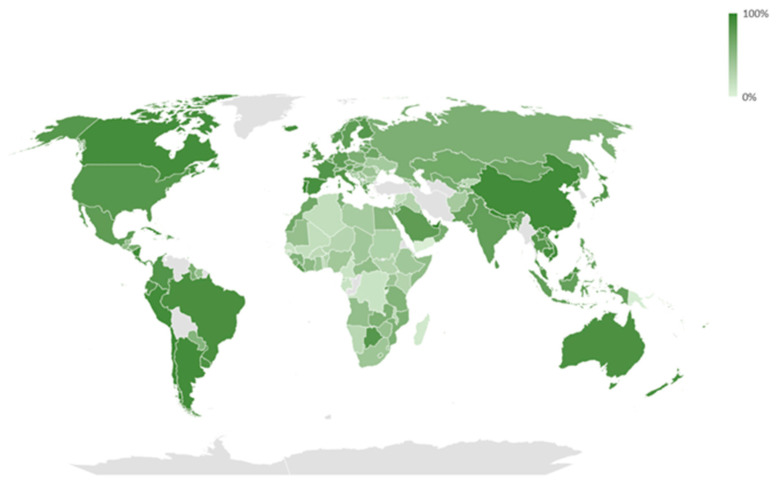
COVID-19 vaccination coverage (until 10 March 2023) in the 178 analyzed countries.

Heterogeneous COVID-19 vaccine coverage was observed (Figure 2), with a median estimate of 64% and an interquartile range spanning from 40% to 82%. According to the consulted dataset, as of 10 March 2023, five countries had vaccination coverages below 10%: Madagascar (8.4%), Papua New Guinea (4.2%), Haiti (3.6%), Yemen (3.4%), and Burundi (0.3%).

### 3.3. All-Cause Excess Mortality Rate and COVID-19 Vaccination Coverage

**Figure 3:**
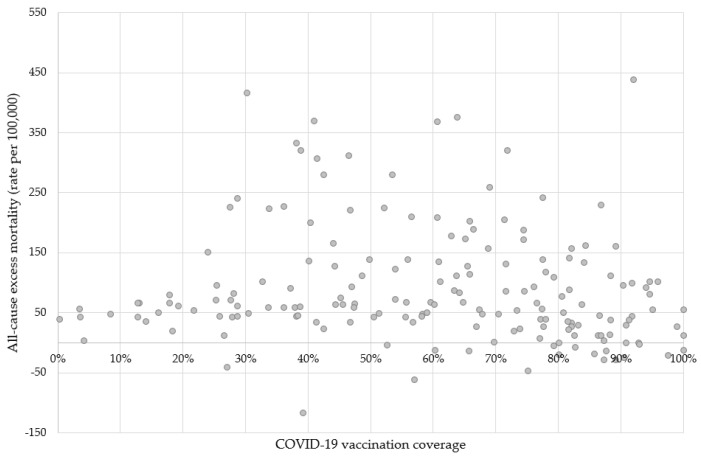
COVID-19 vaccination coverage and all-cause excess mortality rates in the 178 analyzed countries. Note: Vaccination coverage was defined as the proportion of individuals who have received at least one dose of any COVID-19 vaccine.

In the bivariate analysis (Figure 3), a significant negative correlation was observed between the all-cause excess mortality rate and the COVID-19 vaccination coverage (Spearman’s rho = −0.20, 95% CI −0.33 to −0.05, p = 0.009). However, no significant correlation was found between the observed mortality during the study period and life expectancy (p = 0.392).

Our multiple regression analysis revealed a negative correlation between the observed mortality rates and the vaccination coverage (Table 1). Therefore, countries with higher coverage had lower all-cause mortality (β = −106.8, 95% CI −175.4 to −38.2, p = 0.002). Life expectancy at birth also exhibited a significant relationship (β = 3.5, 95% CI 1.2 to 5.8, p = 0.003). Even after excluding the 22 countries with all-cause excess mortality equal to or below 0, the estimates remained significant (COVID-19 vaccination coverage, β = −78.1, 95% CI −148.7 to −7.6, p = 0.030; life expectancy at birth, β = 3.8, 95% CI 1.5 to 6.0, p = 0.002). The adjusted determination coefficients (R^2^) were 0.051 and 0.052 in the models with 178 and 156 countries, respectively.

## 4. Discussion

The findings of our study offer valuable insights into the relationship between all-cause excess mortality and COVID-19 vaccination coverage among the analyzed nations. These findings suggest that countries with higher vaccination coverage experienced lower mortality rates associated with all causes. Similar findings can be found in previously published analyses [9,10]. However, it is important to consider the limitations of our ecological analysis when interpreting these results. In addition, the correlation was relatively weak (Spearman’s rho = −0.20, 95% CI −0.33 to −0.05, p = 0.009), reflecting the complex mechanisms that determined the all-cause excess mortality during the COVID-19 pandemic. Therefore, many other factors, besides vaccination coverage, were not assessed in our study and may have had an undetermined effect on mortality.

The observed median all-cause excess mortality rate of 63 per 100,000 with a wide interquartile range reflects the substantial variations in mortality burden across nations. Notably, several countries demonstrated no positive all-cause excess mortality, indicating effective measures in mitigating excess deaths during the studied period [11]. On the other hand, some countries exhibited significantly higher rates, underscoring the challenges they faced in managing mortality during the COVID-19 pandemic [12].

Our findings, which utilized aggregated and ecological measurements, align with individual-level studies that have demonstrated a correlation between vaccinations and a decreased risk of mortality from severe manifestations of COVID-19 [13]. This protective effect was observed consistently across high-risk population segments [14,15] and varied in timing depending on the specific immunological product administered [16].

Our findings provide valuable insights into the disparities in COVID-19 vaccine coverage. The median estimate of vaccine coverage was determined to be 63%, with an interquartile range spanning from 40% to 82%, indicating significant variation in vaccination efforts among nations. Notably, a subset of countries exhibited alarmingly low vaccination coverages below 10%, underscoring the pressing need to improve vaccine accessibility and distribution in these regions. Immediate attention and support are particularly required for countries with a low vaccination coverage, including Madagascar, Papua New Guinea, Haiti, Yemen, and Burundi, to bolster their immunization initiatives. The factors contributing to the observed situation in these specific countries necessitate careful and individual analysis. For instance, in Madagascar, the COVID-19 Vaccines Global Access (COVAX) initiative was implemented, and the distribution of vaccine doses was based on the elderly population or overall population size in each region [17].

The significant negative correlation between all-cause excess mortality and COVID-19 vaccination coverage highlights the importance of widespread vaccination campaigns in reducing overall mortality [18]. This finding reinforces the crucial role of vaccines in preventing severe outcomes and saving lives during the pandemic. Countries with a higher vaccination coverage exhibited lower all-cause mortality rates, suggesting that immunization efforts may have played a vital role in mitigating the impact of COVID-19 [19].

Additionally, our study identified a significant relationship between life expectancy at birth and observed mortality rates. Higher life expectancy was associated with lower mortality rates, emphasizing the broader implications of population health indicators on mortality outcomes [20,21]. It is important to note that the observed correlation between mortality and life expectancy was independent of the COVID-19 pandemic, suggesting that broader health determinants continue to influence overall mortality rates [22].

As presented in Table 1, the bivariate analysis revealed non-significant coefficients for COVID-19 vaccination coverage (β = −44.6, 95% CI −101.1 to 12.0, p = 0.122). This finding was primarily driven by countries with a low vaccination coverage (below 10%). However, when excluding these nations (n = 5) from the model, the coefficient became statistically significant (β = −68.1, 95% CI −129.4 to −6–8, p = 0.030). Further investigation is required to identify the specific factors in these countries that contribute to the statistical significance of the estimates.

Among the analyzed nations, Peru stands out as a unique case. According to the available datasets, Peru exhibited a significantly high all-cause mortality rate (437 per 100,000) despite achieving a commendable COVID-19 vaccination coverage (92%) [23,24]. This puzzling scenario can be attributed to a multitude of factors. Our hypothesis suggests that these factors encompass the efficacy of vaccines against specific variants, constraints in healthcare capacity, population characteristics, and the execution of public health interventions [25].

Further research is needed to delve into the long-term effects of vaccination coverage on population health amidst the COVID-19 pandemic. Continuous monitoring and investigation will be crucial in comprehending the broader implications of vaccination programs on overall health outcomes. Moreover, evaluating the durability and effectiveness of different vaccine types and examining the potential emergence of new variants remain important areas of investigation.

### Strengths and Limitations

The strength of our findings persisted even after excluding countries with all-cause excess mortality equal to or below zero, indicating the robustness and generalizability of the observed associations. These results underscore the significance of vaccination coverage and population health indicators in shaping mortality outcomes, independent of the absence of excess deaths.

While our study provides important insights, there are certain limitations that should be considered. First, the analysis is based on aggregated data at the country level, which may not capture regional or local variations within countries. These variations may also include factors associated with pandemic waves and prevailing viral variants. Second, the study focused on all-cause excess mortality and did not delve into cause-specific mortality patterns, which could provide further context and understanding. Additionally, the study’s cross-sectional nature limits our ability to establish causal relationships between vaccination coverage, mortality rates, and demographic indicators. Additionally, thirdly, according to the computed correlation, vaccination coverage explained only about 20% of the mortality variation in the analyzed nations. Therefore, many other factors that we omitted influenced pandemic-related all-cause excess mortality.

## 5. Conclusions

Our study highlights the significant negative correlation between all-cause excess mortality and COVID-19 vaccination coverage, supporting the crucial role of vaccination in reducing overall mortality during the pandemic. It emphasizes the importance of widespread vaccine distribution and equitable access to vaccines, particularly in countries with lower coverage rates. Furthermore, our findings underscore the broader implications of population health indicators, such as life expectancy, on mortality outcomes. These insights contribute to the growing body of evidence on the impact of vaccination and population health on overall mortality, informing public health strategies aimed at mitigating the burden of COVID-19 and other causes of death.

## Figures and Tables

**Table 1:** Factors related to all-cause excess mortality from 2020 to 2021.

Variable	β (95% CI), p	
	Bivariate Analysis	Multiple Analysis
COVID-19 vaccination coverage	−44.6 (−101.1 to 12.0), 0.122	−106.8 (−175.4 to −38.2), 0.002
Life expectancy at birth	1.4 (−0.5 to 3.3), 0.147	3.5 (1.2 to 5.8), 0.003

Abbreviations: CI, confidence interval; COVID-19, coronavirus disease 2019. Notes: (1) Linear regression models were employed to derive the presented estimates; (2) the regression coefficients in the multiple model were adjusted for the variables listed in this table.

## Data Availability

The analyzed datasets, can be found using the following URLs: (1) the rates of excess mortality for all causes (per 100,000) associated with the COVID-19 pandemic from 2020 to 2021 were obtained from https://www.who.int/data/sets/global-excess-deaths-associated-with-COVID-19-modelled-estimates (accessed on 15 May 2023); (2) the coverage of COVID-19 vaccinations (percentage of the population that received at least one dose of any COVID-19 vaccine until 10 March 2023) was obtained from https://coronavirus.jhu.edu/vaccines/international (accessed on 15 May 2023); and (3) life expectancies at birth (2021 estimates) were retrieved from https://api.worldbank.org/v2/en/indicator/SP.DYN.LE00.IN?downloadformat=csv (accessed on 15 May 2023).
